# Phthiraptera of Canada

**DOI:** 10.3897/zookeys.819.26160

**Published:** 2019-01-24

**Authors:** Terry D. Galloway

**Affiliations:** 1 Department of Entomology, University of Manitoba, Winnipeg, Manitoba, R3T 2N2, Canada University of Manitoba Winnipeg Canada

**Keywords:** biodiversity assessment, Biota of Canada, lice, Phthiraptera

## Abstract

There are approximately 463 species of parasitic lice recorded in Canada, in three suborders: Amblycera, six families; Ischnocera, two families; Anoplura, eight families. At least an additional 361 species may eventually be recorded based on presence of suitable hosts and proximity to known distributions. Approximately 41 species are introduced non-native species. Only about 54% of the expected chewing louse fauna has been recorded, and considerable collecting effort is needed, especially for lice infesting passerine birds, shorebirds, and seabirds. The sucking louse fauna is well known, with approximately 88% of the expected fauna recorded. Investigations into ecology of lice and the nature of relationships with their hosts are badly needed. Barcode Index Numbers are available for only 13 species of parasitic lice in Canada.

Lice are ubiquitous, obligate external parasites of birds and mammals. At one time, they were treated as two separate orders (see discussion in Palma and Barker 1996), the Mallophaga (chewing lice, parasites of birds and mammals) and the Anoplura (sucking lice, parasites of mammals). They have been consolidated within the order Phthiraptera, divided into four suborders: Anoplura (sucking lice, parasites of mammals), Amblycera, Ischnocera (both chewing lice infesting birds and mammals) and Rhynchophthirina (chewing lice infesting elephants and warthogs, and not known to occur in Canada) (Palma and Barker 1996). Although there is support for combining Psocoptera and Phthiraptera into one order, Psocodea, based on morphological and molecular evidence (Yoshizawa and Johnson 2010, Trautwein et al. 2012), Phthiraptera as an order for parasitic lice is retained here.

Lice have never received a great deal of attention in Canada, and the fauna, especially chewing lice (Amblycera and Ischnocera), is not well known. Based on the compilation of species by Martin (1979), Galloway and Danks (1990) identified chewing lice as one of two highest priority groups of arthropod ectoparasites that warranted particular investigation, and this situation remains unchanged today. There are several studies where lice were collected locally in general surveys (e.g., Twinn 1935, Judd 1953, Teskey 1960, Thompson 1968) or from specific hosts (e.g., Buscher 1965, Judd 1968, Ballard and Ring 1979, Dick 1981, Colwell et al. 2008, Yunik et al. 2016). In regional initiatives, Spencer (1928, 1939, 1948, 1957) collected intensively in British Columbia, and species lists of chewing lice were compiled for Quebec by Rayner (1932) and Whitehead (1934, 1954) and for Alberta by Brown and Wilk (1944). William Threlfall and his students recorded lice from a number of hosts in Newfoundland (e.g., Andrews and Threlfall 1975, Bourgeois and Threlfall 1981, Eveleigh and Threlfall 1974, Fitzpatrick and Threlfall 1977, Threlfall et al. 1979, Threlfall and Wheeler 1986, Wheeler and Threlfall 1986). Galloway et al. (2014) provided a list of species of chewing list infesting grassland birds in Canada. There are a number of recent studies on lice infesting several species of birds in Manitoba (Galloway 2007, 2012, Galloway and Palma 2008, Galloway and Lamb 2014, 2016). The most comprehensive compilations of species of lice found in Canada are those of Wheeler and Threlfall (1989) for birds, and Kennedy (1986) and Kennedy and Newman (1986) for domestic and terrestrial mammals, respectively.

There are many publications to aid identification of lice in Canada. Kim et al. (1986) provided a well-illustrated manual for the identification of the species of Anoplura of North America. With the checklists for the Anoplura and their mammal hosts by Durden and Musser (1994a, b), it should be possible to identify all of the species known to occur in Canada. Unfortunately, there is no such guide to the identification of chewing lice found on birds; keys to the genera are found in Keirans (1966), Ledger (1980), and Price et al. (2003a), but these keys are specialised and not always well illustrated so it takes a considerable length of time to become sufficiently familiar with the terminology to use the keys effectively. Keys to the species of lice on birds are scattered throughout the primary literature, usually focused on species in individual genera, or on species found on selected hosts. No attempt to summarise these is made here. Earlier checklists for the chewing lice by Emerson (1972a, b, c, d), Price and Graham (1997) and Poole (1997a, b) have been eclipsed by the outstanding checklist of Price et al. (2003a). Nomenclature in this latter checklist is adopted here.

There is considerable disagreement about the application of subspecies names to louse taxa. It is assumed that Martin (1979) did not include subspecies as separate taxa in his totals. I have not attempted to address this issue, and therefore ignore all subspecies for the current biodiversity assessment, even where the evidence for their validity is strongly supported (e.g., in some taxa of *Actornithophilus*, *Quadraceps*, and *Saemundssonia*).

There has been little attention on taxonomy at the molecular level for species of lice collected specifically in Canada, with only 13 Barcode Index Numbers (BINs) in the Barcode of Life Data Systems (BOLD) database (Table 1). Grossi et al. (2014) synonymised two species of *Anatoecus* infesting anseriforms in Canada, based on molecular analysis using sequence data from the COI region of mitochondrial DNA.

In compiling the following data on lice in Canada, certain decisions were made about what species should be included. Because lice are permanent ectoparasites of their hosts, they go wherever their hosts go. During winter, many species of birds are far away on their overwintering grounds, so their lice are no longer present in Canada. Many species of birds disperse from their breeding ranges in Eurasia and the United States, for example, and occur in Canada with varied degrees of frequency, though not necessarily to breed. The current list of the louse fauna includes species on such avian hosts, however infrequently they might actually occur within the geographic boundaries of Canada. Therefore, lice from all native and non-native mammals (Banfield 1974) and birds (Godfrey 1986) known to occur in Canada are cited here, including domestic animals and naturalised non-native birds (e.g., rock pigeon, *Columbalivia* Gmelin; European starling, *Sturnusvulgaris* Linnaeus; house sparrow, *Passerdomesticus* (Linnaeus)) and mammals (e.g., house mouse, *Musdomesticus*; Norway rat, *Rattusnorvegicus* Linnaeus). Not all species of lice known to infest non-native introduced hosts are known to occur currently in Canada (Paterson et al. 1999). Some of those may already be present and undetected and others may be introduced in the future (see Galloway and Palma 2008). There is extensive trade and importation of exotic animals into Canada, and although these animals may pass through strict quarantine, it is possible that their lice may initially escape detection. I have made no attempt to compile the records from these exotic species.

Our knowledge of the louse fauna in Canada has only modestly progressed since 1979 (Martin 1979), especially for chewing lice. Currently 463 species of lice, 41 of which are non-native, are known from Canada, 418 of which are chewing lice and only 45 are sucking lice (Table 1). In comparison, Martin (1979) reported 362 species, 329 and 33 of which were chewing and sucking lice, respectively. Although the exact composition of species included by Martin (1979) is not known, he estimated that only 45% of the chewing louse fauna of Canada had been recorded. I estimate about 54% of the chewing lice fauna to be documented (Table 1). In comparison, the sucking lice fauna was believed to have been 94% documented in 1979 (Martin 1979) and 88% documented today (Table 1), the decrease attributed to the fact that the total fauna is now believed to be larger than anticipated in 1979.

There are many specimens of undescribed species in collections and there are many more awaiting discovery. Kim et al. (1990) estimated the numbers of species of lice in North America by extrapolation from known host/parasite associations. I have refrained from adopting their strategy in this paper. In the case of chewing lice on birds, there are a great many host species, especially among the Passeriformes, for which no lice have been recorded. It is not known whether this is because of insufficient collecting, or whether these hosts, in fact, are parasitised by few or no species of lice. Until such gaps are addressed and data are produced, extrapolations based on the known fauna may result in artificially inflated estimates of numbers of taxa.

It is likely that there are many undescribed species of lice, especially in some of the hyperdiverse genera, e.g., *Myrsidea*, *Brueelia* (see Valim and Weckstein 2013, Bush et al. 2016, Gustafsson and Bush 2017). Advanced tools used to explore the molecular basis for species separation should also clarify some relationships among species of lice and add to the growing list of new species. As populations of birds and mammals continue to decline and the numbers of threatened and endangered species increase, many of which are hosts for a great diversity of ectosymbionts, including parasitic lice, it is important that the recommendations of Galloway and Danks (1990) not be forgotten. Of course, having a list of species with relevant geographic and host associations is only the first step in understanding the real nature of the complex relationships among ectoparasites and their hosts.

**Table 1.** Census of Phthiraptera in Canada.

Taxon^1^	No. species reported in Martin (1979)	No. species currently known from Canada^2^	No. BINs^3^ available for Canadian species	Est. no. described but unrecorded species in Canada^4^	General distribution by ecozone^5^ and host range	Information sources^6^
**Suborder Amblycera**						
Menoponidae	100	116 (5)	2	110	all ecozones; birds	Klockenhoff (1984), Martinho Guimarães (1988), Price et al. (2002, 2003a), Price and Dalgleish 2007, Cicchino and González-Acuña (2012), Gustafsson and Olsson (2012); CNCI, WRME
Ricinidae	3	15	1	15	all ecozones but perhaps barren-ground; passerine birds, hummingbirds	Nelson (1972), Price et al. (2003); CNCI, WRME
Laemobothriidae	2	6	0	2	all ecozones; coots, eagles, hawks, falcons	Nelson and Price (1965), Price et al. (2003a); CNCI, WRME
Gyropidae	2	2 (2)	0	1	caviomorph rodents	Price et al. (2003a); CNCI, WRME
Boopiidae	1	1 (1)	0	0	domestic dogs	Price et al. (2003a); CNCI, WRME
Trimenoponidae **^7^**	1	0	0	0		Price et al. (2003a); CNCI, WRME
**Suborder Ischnocera**						
Philopteridae	200	250 (17)	6	223	all ecozones; birds	Cicchino (1980), Eichler and Vasjukova (1981), Balát (1982), Mey (1982), Price et al. (2003a, b), Gustafsson and Olsson (2012), Gustafsson and Bush (2017); CNCI, WRME
Trichodectidae	20	31 (7)	1	4	all ecozones; mammals	Hopkins (1960), Lyal (1985), Price et al. (2003a); CNCI, WRME
**Suborder Anoplura**						
Echinophthiriidae	3	6	0	0	widespread in marine and estuarine habitats; marine mammals	Kim et al. (1986), Durden and Musser (1994a); CNCI, WRME
Enderleinellidae **^8^**	?	5	0	0	all ecozones; small mammals	Kim et al. (1986), Durden and Musser (1994a); CNCI, WRME
Polyplacidae **^8^**	?	13 (1)	1	3	all ecozones; small mammals	Kim et al. (1986), Durden and Musser (1994a); CNCI, WRME
Haematopinidae	3	3 (3)	0	1	all ecozones; hoofed mammals, including domestic animals	Kim et al. (1986), Durden and Musser (1994a); CNCI, WRME
Hoplopleuridae **^8^**	19	7	1	1	all ecozones; small mammals	Kim et al. (1986), Durden and Musser (1994a); CNCI, WRME
Linognathidae	6	9 (5)	0	1	most ecozones; hoofed mammal and canids, including domestic animals	Kim et al. (1986), Durden and Musser (1994a); CNCI, WRME
Pediculidae	1	1	1	0	all ecozones; humans	Kim et al. (1986), Durden and Musser (1994a); CNCI, WRME
Pthiridae	1	1	0	0	all ecozones; humans	Kim et al. (1986), Durden and Musser (1994a); CNCI, WRME
**Total**	**362**	**463 (41)**	**13**	**361**		

**^1^**Classsifcation follows that of Price et al. (2003) for chewing lice and Durden and Musser (1994a) for sucking lice. **^2^**The numbers in parentheses represents the number of non-native species included in the total. **^3^**Barcode Index Number, as defined in Ratnasingham and Hebert (2013). **^4^**No attempt was made to include undescribed species that may occur in Canada. **^5^**See figure 1 in Langor (2019) for a map of ecozones. **^6^**References are relevant to species described since 1979 and are known or suspected to occur in Canada. WRME – Wallis/Roughley Museum of Entomology, University of Manitoba, Winnipeg, Manitoba; CNCI – Canadian National Collection of Insects, Arachnids, and Nematodes, Ottawa, Ontario. **^7^**Martin (1979) listed one species of Trimenoponidae in his table. There are no endemic species of this family in Canada. The only record I can find of a trimenoponid in the CNCI, for *Harrisoniauncinata* Ferris, 1922, is from Trinidad. This family is not included in the current list. **^8^**Martin (1979) presumably included the Enderleinellidae and Polyplacidae as subfamilies of Hoplopleuridae, by inference from his reference to Ferris (1951). Since Martin provided no tally of species included in each subfamily, only his total number of species for Hoplopleuridae is presented here.

## References

[B1] Andrews SE Threlfall W (1975) Parasites of the common crow (*Corvusbrachyrhynchos* Brehm, 1822) in insular Newfoundland. Proceedings of the Helminthological Society of Washington 42: 24–28.

[B2] Balát F (1982) Zwei neue Federlinge (Mallophaga) aus Serrahn. Zoologischer Rundbrief für den Bezirk Neubrandenberg 1982: 43–47.

[B3] Ballard JT Ring RA (1979) The ectoparasites of some marine birds from Bamfield Marine Station, British Columbia, with particular reference to the common murre (*Uriaaalge* (Pont.)). Canadian Journal of Zoology 57: 1980–1984. 10.1139/z79-262

[B4] Banfield AWF (1974) The Mammals of Canada. National Museum of Natural Sciences, National Museums of Canada, University of Toronto Press, Toronto, xxv + 438 pp.

[B5] Bourgeois CE Threlfall W (1981) Mallophaga from three species of scoters (Anatidae). Proceedings of the Entomological Society of Washington 83: 799–800.

[B6] Brown JH Wilk AL (1944) Mallophaga of Alberta: a list of species with hosts. Canadian Entomologist 76: 127–129. 10.4039/Ent76127-6

[B7] Buscher HN (1965) Ectoparasites from anseriform birds in Manitoba. Canadian Journal of Zoology 43: 219–221. 10.1139/z65-020 14287037

[B8] Bush SE Weckstein JD Gustafsson DR Allen JM DiBlasi E Shreve SM Boldt R Skeen HR Johnson KP (2016) Unlocking the black box of feather louse diversity: a molecular phylogeny of the hyper-diverse genus *Brueelia*. Molecular Phylogenetics and Evolution 94: 737–751. 10.1016/j.ympev.2015.09.015 26455895

[B9] Cicchino AC (1980) Contribucion al conocimiento de los malófagos y algunos malófagos hallados en *Troglodytesaedonbonariae* Hellmayr en la Provincia de Buenos Aires. Revista de al Sociedad Entomological Argentina 39: 5–10.

[B10] Cicchino AC González-Acuña DA (2012) Species in the genus *Bonomiella* Conci, 1942 (Phthiraptera: Menoponidae) from Argentina and Chile. Zootaxa 3427: 47–56.

[B11] Colwell DD Gray D Morton K Pybus M (2008) Nasal bots and lice from white-tailed deer in southern Alberta, Canada. Journal of Wildlife Diseases 44: 687–692. 10.7589/0090-3558-44.3.687 18689654

[B12] Dick TA (1981) Ectoparasites of sharp-tailed grouse, *Pediocetesphasianellus*. Journal of Wildlife Diseases 17: 229–235. 10.7589/0090-3558-17.2.229 7241708

[B13] Durden LA Musser GG (1994a) The sucking lice (Insecta: Anoplura) of the world: a taxonomic checklist with records of mammalian hosts and geographical distributions. Bulletin of the American Museum of Natural History No. 218, 90 pp.

[B14] Durden LA Musser GG (1994b) The mammalian hosts of the sucking lice (Anoplura) of the world: a host-parasite list. Bulletin of the Society for Vector Ecology 19: 130–168.

[B15] Eichler W Vasjukova TT (1981) A new species of the genus *Docophorus* Eichler (Mallophaga, Philopteridae) from the white-winged crossbill *Loxialeucopterabifasciata*, Brehm. Entomologicheskoe Oborzrenie 60: 620–622.

[B16] Emerson KC (1972a) Checklist of the Mallophaga of North America (North of Mexico) Part I. Sub-order Ischnocera. Deseret Test Center, Dugway, Utah, 200 pp.

[B17] Emerson KC (1972b) Checklist of the Mallophaga of North America (North of Mexico) Part II. Sub-order Amblycera. Deseret Test Center, Dugway, Utah, 118 pp.

[B18] Emerson KC (1972c) Checklist of the Mallophaga of North America (North of Mexico) Part III. Mammal host list. Deseret Test Center, Dugway, Utah, 28 pp.

[B19] Emerson KC (1972d) Checklist of the Mallophaga of North America (North of Mexico). Part IV. Bird host list. Deseret Test Center, Dugway, Utah, 216 pp.

[B20] Eveleigh ES Threlfall W (1974) A new species, and notes on a previously described species of *Austromenopon* Bedford, 1939 (Mallophaga: Amblycera) from alcids (Aves: Charadriiformes). Proceedings of the Entomological Society of Washington 76: 270–277.

[B21] Ferris GF (1951) The sucking lice. Memoirs of the Pacific Coast Entomological Society, Vol. 1: ix + 320 pp. 10.5962/bhl.title.149669

[B22] Fitzpatrick C Threlfall W (1977) The ectoparasites of three species of seabirds from Newfoundland, Canada. Canadian Journal of Zoology 55: 1205–1209. 10.1139/z77-158 884643

[B23] Galloway TD (2007) Ectoparasites (Phthiraptera: Philopteridae; Acari: Ixodidae) of Common Nighthawk, *Chordeilesminor*, and Whip-poor-will, *Caprimulgusvociferus* (Caprimulgiformes: Caprimulgidae), in Manitoba. Journal of the Entomological Society of Ontario 137: 2–9.

[B24] Galloway TD (2012) Ectoparasites of rabbits and hares (Lagomorpha: Leporidae) in Manitoba, with observations on age-specific dispersal in the sucking louse, *Haemodipsussetoni* (Phthiraptera: Anoplura: Polyplacidae). The Canadian Entomologist 144: 439–446. 10.4039/tce.2012.44

[B25] Galloway TD Danks HV (1990) Arthropod ectoparasites of vertebrates in Canada. A brief prepared by the Biological Survey of Canada. Bulletin of the Entomological Society of Canada 23 (1991), 11 pp.

[B26] Galloway TD Lamb RJ (2014) Abundance and stability are species traits for four chewing lice (Phthiraptera: Menoponidae, Philopteridae) on feral pigeons, *Columbalivia* Gmelin (Aves: Columbiformes: Columbidae). The Canadian Entomologist 146: 444–456. 10.4039/tce.2013.86

[B27] Galloway TD Lamb RJ (2016) Chewing lice (Phthiraptera: Amblycera and Ischnocera) infesting woodpeckers, flickers and sapsuckers (Aves: Piciformes: Picidae) in Manitoba, Canada. The Canadian Entomologist 148: 520–531. 10.4039/tce.2015.89

[B28] Galloway TD Palma RL (2008) Serendipity with chewing lice (Phthiraptera: Menoponidae, Philopteridae) infesting rock pigeons, *Columbalivia*, and mourning doves, *Zenaidamacroura*, (Aves: Columbiformes: Columbidae) in Manitoba, with new records for North America and Canada. The Canadian Entomologist 140: 208–218. 10.4039/n07-041

[B29] Galloway TD Proctor HC Mironov SV (2014) Chapter 5. Chewing lice (Insecta: Phthiraptera: Amblycera, Ischnocera) and feather mites (Acari: Astigmatina: Analgoidea, Pterolichoidea): ectosymbionts of grassland birds in Canada. In: Cárcamo H Giberson DJ (Eds) Arthropods of Canadian Grasslands, Volume 3.Biodiversity and Systematics, Part 1. Biological Survey of Canada, Ottawa, Ontario, 139–188.

[B30] Godfrey WE (1986) The Birds of Canada. Revised Edition. National Museum of Natural Sciences, National Museums of Canada, Ottawa, Ontario, 595 pp.

[B31] Grossi AA Sharanowski BJ Galloway TD (2014) *Anatoecus* species (Phthiraptera: Philopteridae) from Anseriformes in North America and taxonomic status of *Anatoecusdentatus* and *Anatoecusicterodes*. The Canadian Entomologist 146: 598–608. 10.4039/tce.2014.12

[B32] Gustafsson DR Olsson U (2012) The “very thankless task”: revision of *Lunaceps* Clay & Meinertzhagen, 1939 (Insecta: Phthiraptera: Ischnocera: Philopteridae), with descriptions of six new species and one new subspecies. Zootaxa 3377: 1–85.

[B33] Gustafsson DR Bush SE (2017) Morphological revision of the hyperdiverse *Brueelia*-complex (Insecta: Phthiraptera: Ischnocera: Philopteridae) with new taxa, checklists and generic key. Zootaxa 4313: 1–443. 10.11646/zootaxa.4313.1.1

[B34] Hopkins GHE (1960) Notes on some Mallophaga from mammals. Bulletin of the British Museum (Natural History). Entomology 10: 77–95 + 2 plates.

[B35] Judd WW (1953) A collection of feather lice (Mallophaga) from birds in Ontario. Transactions of the American Microscopical Society 72: 349–350. 10.2307/3223482

[B36] Judd WW (1968) Ectoparasites from the cowbird, *Molothrusater* (Boddaert), at London, Ontario. Canadian Journal of Zoology 46: 807. 10.1139/z68-113 5749477

[B37] Keirans JE (1966) The Mallophaga of New England birds. PhD thesis, University of New Hampshire, New Hampshire, x + 428 pp.

[B38] Kennedy MJ (1986) Synopsis of parasites of domesticated mammals of Canada. Alberta Agriculture, Animal Health Division, Edmonton, Alberta, 53 pp 10.5962/bhl.title.111601

[B39] Kennedy MJ Newman RA (1986) Synopsis of parasites of vertebrates of Canada. Ectoparasites of terrestrial mammals. Alberta Agriculture, Animal Health Division, Edmonton, Alberta, 109 pp 10.5962/bhl.title.110489

[B40] Kim KC Emerson KC Traub R (1990) Diversity of parasitic insects: Anoplura, Mallophaga, and Siphonaptera. In: Kosztarab M Schaefer CW (Eds) Systematics of the North American insects and arachnids: status and needs.Virginia Agricultural Experiment Station Information Series 90-1. Virginia Polytechnic Institute and State University, Blacksburg, 91–103.

[B41] Kim KC Pratt HD Stojanovich CJ (1986) The sucking lice of North America. An illustrated manual for identification. Pennsylvania State University Press, 241 pp.

[B42] Klockenhoff H (1984) *Myrsidealyali* n. sp. (Phthiraptera: Menoponidae) ein neuer Federling von *Fringillacoelebs* (Passeriformes: Fringillidae). Bonner zoologischer Beiträge 35: 263–268.

[B43] Langor DW (2019) The diversity of terrestrial arthropods in Canada. In: Langor DW Sheffield CS (Eds) The Biota of Canada – A Biodiversity Assessment. Part 1: The Terrestrial Arthropods.ZooKeys 819: 9–40. 10.3897/zookeys.819.31947 PMC6355749 30713431

[B44] Ledger JA (1980) The arthropod parasites of vertebrates in Africa south of the Sahara. Volume IV. Phthiraptera (Insecta). South African Institute for Medical Research, Johannesburg, iv + 327 pp.

[B45] Lyal CHC (1985) A cladistic analysis and classification of trichodectid mammal lice (Phthiraptera: Ischnocera). Bulletin of the British Museum (Natural History), Entomology Series 51: 187–346.

[B46] Martin JEH (1979) 34. Phthiraptera (Mallophaga and Anoplura). In: Danks HV (Ed.) Canada and its insect fauna.Memoirs of the Entomological Society of Canada No. 108, 326–328. 10.4039/entm111108326-1

[B47] Martinho Guimarães AM (1988) Uma nova espécie de *Actornithophilus* Ferris (Mallophaga, Menoponidae). In: Actas III Congreso Ibérico de Entomologia, Granada, Spain, 157–162.

[B48] Mey E (1982) Mongolische Mallophagen II. (Ergebnisse der Mongolische-Deutschen Biologische Expeditionen seit 1962, Nr. 111). Reichenbachia 20: 59–65.

[B49] Nelson BC (1972) A revision of the new world species of *Ricinus* (Mallophaga) occurring on Passeriformes (Aves). University of California Publications in Entomology 68: 1–130. [+ 43 plates]

[B50] Nelson RC Price RA (1965) The *Laemobothrion* (Mallophaga: Laemobothriidae) of the Falconiformes. Journal of Medical Entomology 2: 249–257. 10.1093/jmedent/2.3.249

[B51] Palma RL Barker SC (1996) Phthiraptera. In: Wells A (Ed.) Zoological Catalogue of Australia.Volume 26. Psocoptera, Phthiraptera, Thysanoptera. CSIRO Publishing, Melbourne, 81–88.

[B52] Paterson AM Palma RL Gray RD (1999) How frequently do avian lice miss the boat? Implications for coevolutionary studies. Systematic Biology 48: 214–223. 10.1080/106351599260544

[B53] Poole RW (1997a) Anoplura. In: Poole RW Gentili P (Eds) Nomina Insecta Nearctica: a check list of the insects of North America.Volume 4. Non-holometabolous orders. Entomological Information Services, Rockville, Maryland, 19–26.

[B54] Poole RW (1997b) Mallophaga. In: Poole RW Gentili P (Eds) Nomina Insecta Nearctica: a check list of the insects of North America.Volume 4. Non-holometabolous orders. Entomological Information Services, Rockville, Maryland, 497–544.

[B55] Price MA Graham OH (1997) Chewing and sucking lice as parasites of mammals and birds. United States Department of Agriculture, Agricultural Research Service. Technical Bulletin No. 1849, 257 pp. + 2 appendices.

[B56] Price RD Dalgleish RC (2007) *Myrsidea* Waterston (Phthiraptera: Menoponidae) from the Emberizidae (Passeriformes), with descriptions of 13 new species. Zootaxa 1467: 1–18. 10.1674/0003-0031(2002)148[0061:AROMPA]2.0.CO;2

[B57] Price RD Hellenthal RA Dalgleish RC (2002) A review of *Machaerilaemus* (Phthiraptera: Amblycera: Menoponidae) from the Passeriformes (Aves), with the description of five new species. American Midland Naturalist 148: 61–74.

[B58] Price RD Hellenthal RA Palma RL (2003a) World checklist of chewing lice with host associations and keys to families and genera. In: Price RD Hellenthal RA Palma RL Johnson KP Clayton DH (Eds) The chewing lice: world checklist and biological overview.Illinois Natural History Survey Special Publication 24, 1–448.

[B59] Price RD Palma RL Clayton DH (2003b) Review of the genus *Saemundssonia* Timmermann (Phthiraptera: Philopteridae) from the Alcidae (Aves: Charadriiformes), including a new species and new host records. Proceedings of the Entomological Society of Washington 105: 915–924.

[B60] Ratnasingham S Hebert PDN (2013) A DNA-based registry for all animal species: the Barcode Index Number (BIN) system. PLoS ONE 8(7): e66213. 10.1371/journal.pone.0066213 PMC3704603 23861743

[B61] Rayner JA (1932) Parasites of wild birds in Quebec. Scientific Agriculture 12: 307–309.

[B62] Spencer GJ (1928) External parasites on certain birds in British Columbia. The Canadian Entomologist 60: 257–260. 10.4039/Ent60257-11

[B63] Spencer GJ (1939) Ectoparasites of birds and mammals in British Columbia. V. Parasites of domestic animals (Mammals). Proceedings of the Entomological Society of British Columbia 35: 19–23.

[B64] Spencer GJ (1948) Some records of Mallophaga from British Columbia birds. Proceedings of the Entomological Society of British Columbia 44: 3–6.

[B65] Spencer GJ (1957) Further records of Mallophaga from British Columbia birds. Proceedings of the Entomological Society of British Columbia 53: 3–10.

[B66] Teskey HJ (1960) Survey of insects affecting livestock in southwestern Ontario. The Canadian Entomologist 92: 531–544. 10.4039/Ent92531-7

[B67] Thompson RP (1968) A survey of ectoparasite infestations on poultry flocks in Nova Scotia and Prince Edward Island. The Canadian Entomologist 100: 402–407. 10.4039/Ent100402-4

[B68] Threlfall W Bourgeois CE Bain GA (1979) Mallophaga from some North American Anatidae. Proceedings of the Entomological Society of Washington 81: 327–328.

[B69] Threlfall W Wheeler TA (1986) Ectoparasites from birds in Newfoundland. Journal of Wildlife Diseases 22: 273–275. 10.7589/0090-3558-22.2.273 3712656

[B70] Trautwein MD Wiegmann BM Beutel R Kyer KM Yeates DK (2012) Advances in insect phylogeny at the dawn of the postgenomic era. Annual Review of Entomology 57: 449–468. 10.1146/annurev-ento-120710-100538 22149269

[B71] Twinn CR (1935) Records of Mallophaga and other ectoparasites from birds at Churchill, Manitoba. The Canadian Entomologist 67: 157–159. 10.4039/Ent67157-7

[B72] Valim MP Weckstein JD (2013) A drop in the bucket of the megadiverse chewing louse genus *Myrsidea* (Phthiraptera, Amblycera, Menoponidae): ten new species from Amazonian Brazil. Folia Parasitica 60: 377–400. 10.14411/fp.2013.040 24471279

[B73] Wheeler TA Threlfall W (1986) Observations on some ectoparasites of some Newfoundland passerines (Aves: Passeriformes). Canadian Journal of Zoology 64: 630–636. 10.1139/z86-093

[B74] Wheeler TA Threlfall W (1989) Ectoparasites of birds. In: Kennedy MJ (Ed.) Synopsis of the parasites of vertebrates in Canada.Alberta Agriculture, Animal Health Division, Edmonton, Alberta, 1–85.

[B75] Whitehead WE (1934) Records of some Quebec Mallophaga and Anoplura. Quebec Society for Protection of Plants Report 25–26: 84–87.

[B76] Whitehead WE (1954) Avian Mallophaga from Quebec. The Canadian Entomologist 86: 65–68. 10.4039/Ent8665-2

[B77] Yoshizawa K Johnson KP (2010) How stable is the “polyphyly of lice” hypothesis (Insecta: Psocodea)? A comparison of phylogenetic signal in multiple genes. Molecular Phylogenetics and Evolution 55: 939–351. 10.1016/j.ympev.2010.02.026 20211746

[B78] Yunik MEM Waterman JM Galloway TD (2016) Seasonal changes in the infestation parameters of the sucking louse, *Linognathoideslaeviusculus* (Phthiraptera: Anoplura: Polyplacidae), infesting Richardson’s ground squirrel (Rodentia: Sciuridae) in Manitoba. The Canadian Entomologist 148: 143–150. 10.4039/tce.2015.49

